# Xenotransplantation of Cryopreserved Calf Testicular Tissues

**DOI:** 10.3390/vetsci12030247

**Published:** 2025-03-04

**Authors:** Yansen Zhao, Wenqian Zhu, Rui Yang, Boyang Zhang, Bo Tang, Xueming Zhang

**Affiliations:** State Key Laboratory for Diagnosis and Treatment of Severe Zoonotic Infectious Diseases, College of Veterinary Medicine, Jilin University, Changchun 130062, China; yszhao21@mails.jlu.edu.cn (Y.Z.); zhuwq21@mails.jlu.edu.cn (W.Z.); ruiyang22@mails.jlu.edu.cn (R.Y.); zby23@mails.jlu.edu.cn (B.Z.); tang_bo@jlu.edu.cn (B.T.)

**Keywords:** cattle, freezing–thawing, mouse recipient, testis, xenograft

## Abstract

The cryopreservation of testicular tissues meets the demands for the germplasm preservation of humans and animals. Previously, we reported on the cryopreservation of bovine testicular tissues. To further evaluate the viability of these tissues, subcutaneous xenotransplantation of the frozen–thawed calf testicular tissues was performed with castrated nude mice as the recipients. After 28 days (D28), the survival and development of the grafts were examined. The grafts from 1-day-old (D1) calf testes were recovered and angiogenesis around the grafts was observed. Histologically, the seminiferous cords in the grafts were well maintained and capillaries in the interstitium were observed. Quantitative real-time PCR (qRT-PCR) analysis showed that the grafts expressed germline genes *Gfrα-1*, *C-kit*, and *Sycp3* and somatic genes *Sox9*, *Acta2,* and *Star*. The expressions of *C-kit*, *Sox9*, *Acta2*, and Star were higher in 28D grafts than those in 1D and 30-day-old (30D) calf testicular controls. Together, we initially demonstrate that cryopreserved calf testicular tissues retain their viability and developmental capacity after xenotransplantation.

## Simple Summary

The cryopreservation of testicular tissues is one of the strategies used for the germplasm preservation of endangered animals and especially to ensure high-quality and high-output livestock. The isolation of live cells and tissue transplantation are generally used to test the viability of cryopreserved tissues. Previously, we developed the freezing protocol of bovine testicular tissues and demonstrated the viability of these frozen–thawed bovine testicular tissues by the isolation and culture of viable cells. However, the whole viability of these tissues still needs to be verified. For this purpose, the subcutaneous xenotransplantation of calf testicular tissues was performed with castrated nude mice as recipients. Subsequently, the survival and development of the grafts were observed and analyzed initially after 28 days, which showed that cryopreserved calf testicular tissues retain their viability and developmental capacity after thawing and xenotransplantation.

## 1. Introduction

Cryopreserved testicular tissues have a wide range of applications, particularly for the germplasm preservation of humans and animals [1,2,3]. With the continuous improvements in cryopreservation technology, combined with optimized culture systems and transplant techniques, the cryopreservation of testicular tissues lays a foundation for the further study of the spermatogenesis, diagnosis, and treatment of male sterility [2,3,4,5]. Generally, viability of the frozen–thawed testicular tissues can be assayed by three methods: (1) isolate and culture viable cells, (2) culture tissue fragments/blocks, and (3) transplant whole tissue fragments/blocks. The third method might be the better protocol to evaluate the histological integrity and entire viability of the frozen–thawed testicular tissues.

Tissue transplantation includes autotransplantation, allotransplantation, and xenotransplantation. Testicular tissue transplantation is an effective strategy to study the mechanism of spermatogenesis and treat male sterility. The autotransplantation of testicular tissues can be conducted orthotopically or heterotopically. The ability of cells to differentiate and form functional sperm is closely related to the status of immature testicular tissues. Healthy offspring were successfully obtained by the autotransplantation of immature testicular tissue from mice [1,6], rabbit [6], pig [1,7,8,9,10], and non-human primates [11,12], combined with intracytoplasmic monosperm microinjection. These reports confirmed the application feasibility of the cryopreservation of testicular tissue and subsequent transplantation in reproduction. Testicular xenotransplantation mostly uses mice as recipients, particularly for studies in large animals, such as pig [1,7,8,9,10], goat [1], sheep [13,14], buffalo [15], and non-human primate macaques [11,12]. In cattle, the ectopic xenografting of fresh testicular tissues has also been reported [16,17,18,19,20]. However, whether frozen–thawed bovine testicular tissues can survive and develop after xenotransplantation is unknown.

Based on our previous work [20,21], cryopreserved testicular tissues from 1-day-old (D1) calves were ectopically xenografted under the skin on the backs of castrated nude mice, and the grafts 28 days (D28) after transplantation were evaluated for growth, angiogenesis, and gene expressions, with the ungrafted testicular tissues of D1 and 30-day-old (D30) calves used as controls. We briefly showed here that the cryopreserved calf testicular tissues remained their integrity and viability in vivo after xenotransplantation, which provides evidence for the future application of cryopreserving–grafting testicular tissues in large livestock.

## 2. Materials and Methods

### 2.1. Bovine Testes and Chemicals

The testicular tissues of D1 and D30 calves (n = 3, respectively) were all cryopreserved and thawed as described [20,21]. Nine BALB/c (a laboratory white mouse breed, named by Halsey J. Bagg. He mixed his name “Bagg” and the word “albino”. “c” is the recessive epistatic gene for white coat color) male nude mice aged 4–5 weeks (14–16 g) were used as the recipients (Liaoning Changsheng Biotechnology Co., Ltd., Benxi, Liaoning Province, China). The experimental materials and procedures used here received endorsement from the Animal Welfare and Research Ethics Committee at Jilin University (No. SY201903002). Chemicals including matrigel (Gibco, Vacaville, CA, USA), 2% pentobarbital sodium (Beijing Chemical Works, Beijing, China), phosphate-buffered saline (PBS, BOSTER, Wuhan, China), and 4% paraformaldehyde solution (Biosharp, Hefei, China) were used. TransScript^®^ All-in-One First-Strand cDNA Synthesis SuperMix was sourced from TransGen Biotech (Beijing, China).

### 2.2. Experimental Design, Recipient Castration, and Tissue Xenografting

Experimental Design: Nine mice were all castrated as the recipients, which all received grafts of D1 calf testicular tissues. The ungrafted testicular tissues of D1 and D30 calves (cryopreserved and thawed) were used as controls for a histological comparison and mRNA level analysis.

Recipient Castration: Each mouse was kept in a supine position after it was anesthetized by intraperitoneal injection with 2% pentobarbital sodium (40 mg/kg body weight). A 0.5 cm incision was made in the scrotum and the testes were removed. The spermatic cords and fat pads were returned to the abdominal cavity, following which the peritoneum and skin were sutured closed using medical suture.

Tissue Xenografting: During the surgery, each mouse received three incisions (~3 mm) on each side of the back, as described [15]. The cryopreserved D1 calf testicular tissues were thawed in a water bath at 37 °C and cut into pieces of 1–2 mm^3^. One piece of tissue was inserted through each incision, and each incision was injected with 30 μL of Matrigel. In total, each recipient received 6 pieces of the tissues. The incisions were disinfected and sutured closed. After 28 days, the grafts were collected for subsequent histological and gene expression analysis.

### 2.3. Hematoxylin and Eosin (HE) Staining

The tissues were fixed with PBS 4% formaldehyde for 24 h and embedded in paraffin wax. Each sample was cut into 5 μm thick sections. They were deparaffinized and rehydrated with a series of ethanol and then stained with hematoxylin and eosin. Histological observation was performed.

### 2.4. Quantitative Real-Time Polymerase Chain Reaction (qRT-PCR)

RNA was isolated from the sample tissues (D1 control, D28 grafts, and D30 controls) using TRIzon reagent (CoWin Biosciences, Jiangsu, China). Isolated RNAs were reverse-transcribed by Trans-Script^®^ All-in-One First-Strand cDNA Synthesis SuperMix (TransGen, Beijing, China) to cDNA. qRT-PCR was used to evaluate the relative mRNA levels of germline genes (*Gfrα-1*, *C-kit*, *Sycp3*) and somatic genes (*Sox9*, *Acta2*, *Star*). The primers are listed in Table 1. The reactions were performed in a 20 μL volume system. The program used for gene amplification adhered to the guidelines provided by the kit manufacturer. *GAPDH* was set as the reference gene, and the 2^−ΔΔCt^ method was applied for the analysis of relative gene expression.

### 2.5. Statistical Analysis

The quantitative experiments were performed in triplicate. Data were presented as mean ± standard errors (SD). SPSS 22.0 and GraphPad Prism 8.3.0 software were applied for statistics. One-way ANOVA was used for significance analysis, and * *p* < 0.05 indicates a significant difference.

## 3. Results

### 3.1. Surgery and Angiogenesis in the Xenografts

**Figure 1:**
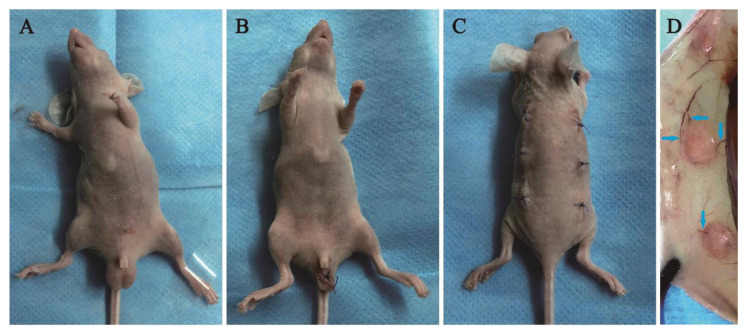
Calf testicular tissues were transplanted on the back of castrated Balb/c male nude mice. (**A**) Anesthesia; (**B**) gonadectomy; (**C**) transplantation of D1 calf testicular tissues and suture; (**D**) grafts on D28; note the blood vessels around grafts (blue arrows).

Xenotopic transplantation of the frozen–thawed tissues is shown in Figure 1. After general anesthesia (Figure 1A) and castration (Figure 1B), the D1 testicular tissues were ectopically xenografted under the skin on the backs of the recipients (Figure 1C). The growth of the grafts was visually identified during the grafting period. After 28 days, all the grafts were enveloped by loose connective tissue, and clear vascular networks were observed around them (Figure 1D). They were collected for the following analysis.

### 3.2. Histological Examination of the Xenografts

**Figure 2:**
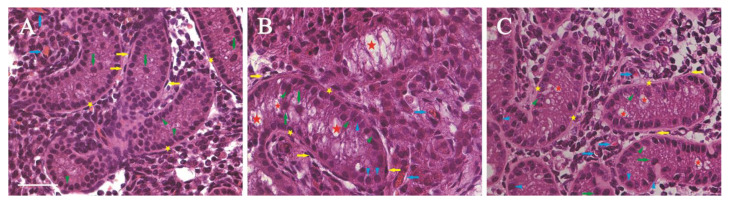
HE staining of the transplanted calf testicular tissues. (**A**) Ungrafted D1 calf testicular tissues; (**B**) D28 xenografted tissues; (**C**) ungrafted D30 calf testicular tissues. Green arrows: gonocytes, blue triangles: spermatogonial stem cells (SSCs), green triangles: Sertoli cells (SCs), yellow arrows: peritubular myoid cells (PMCs), red stars: vacuoles, yellow stars: basement membrane, blue arrows: capillaries. Scale bar = 50 μm in (**A**–**C**).

Histological analysis of the tissue architecture, structural integrity, and cellular morphology was performed. Compared with the D1 control tissues (Figure 2A), microstructures of the seminiferous cords, including gonocytes/spermatogonial stem cells (SSCs), immature Sertoli cells (SCs), basement membrane, and peritubular myoid cells (PMCs), in the D28 grafts were well maintained, and the cord diameters slightly increased (Figure 2B). In the D28 graft interstitium (Figure 2B), capillaries were also observed. They are similar with those in D1 and D30 control tissues (Figure 2A–C). Gradually, vacuoles appeared in the center of the seminiferous cords in the D28 grafts, which are similar with those in the D30 tissues (Figure 2C).

### 3.3. Gene Expressions in the Xenografts

**Figure 3:**
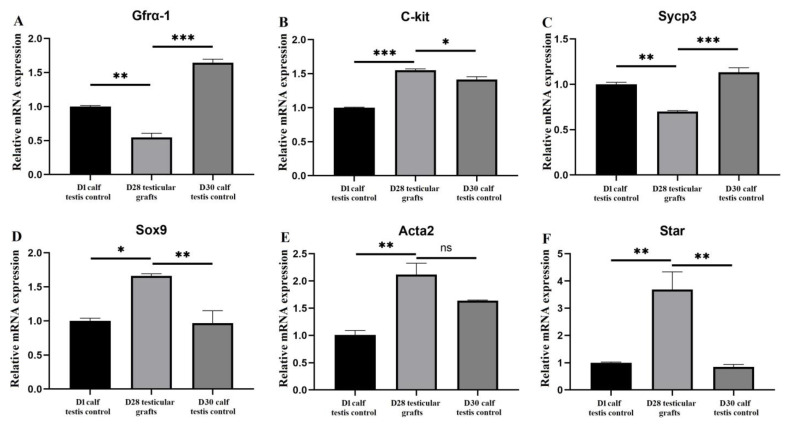
qRT-PCR analysis of the gene expressions in the xenografts. (**A**) SSC marker *Gfrα-1*; (**B**) spermatogenic differentiating gene *C-kit*; (**C**) meiotic gene *Sycp3*; (**D**) SC marker *Sox9*; (**E**) PMC marker *Acta2*; (**F**) Leydig cell marker *Star*; data were presented as mean ± SD, * *p*  <  0.05, ** *p*  <  0.01, *** *p*  <  0.001, and ns = no statistical significance.

Next, transcriptions of the SSC marker gene *Gfrα-1*, differentiating spermatogenic gene *C-kit*, and meiosis gene *Sycp3* were analyzed. The mRNA levels of *Gfrα-1* in the D28 grafts were lower than those both in the D1 and D30 controls (Figure 3A). In contrast, an increased mRNA level of the *C-kit* was detected in the D28 grafts compared to those in the controls (Figure 3B). For *Sycp3*, a similar expression trend was observed as for *Gfrα-1* (Figure 3C). To further investigate the graft development, the mRNA levels of SC marker *Sox9*, PMC marker *Acta2*, and Leydig cell marker *Star* were assayed, showing increased expressions of *Sox9*, *Acta2,* and *Star* in D28 grafts (*p* < 0.05 or 0.01 except *Acta2* in D28 grafts vs. D30 control, Figure 3D–F).

## 4. Discussion

Cryopreserved testicular tissues can be used to isolate and culture SSCs or perform tissue culture. Previously, we had reported the isolation and culture of gonocytes/SSCs from frozen–thawed bovine testicular tissues [20,21]. These materials can also be cultured as tissue fragments/blocks, but it is difficult to preserve their histological structure in vitro. Compared with germ cell culture and testicular tissue culture, the transplantation of frozen–thawed testicular tissues is able to preserve the tissue structure, particularly the spermatogenic environments/niches, and determine the entire biological activity of these tissues.

Although testicular tissue transplantation was reported a decade ago and the donor germ cells can develop and differentiate into sperm in several species, some problems/questions still need to be solved for its further application. Previously, the transplantation of cryopreserved testicular pieces from several species was reported [6,22,23]. However, the survival and development possibility of frozen–thawed bovine testicular tissues after transplantation is unknown. Studies have shown that the serum testosterone concentration in normal newborn males is very low, and it does not reach a high level until late spermatogenesis [15]. Thus, castrated adult immunodeficient mice were used as recipients here to simulate the low-concentration testosterone environment. Additionally, circulation establishment between the grafts and recipients is key to the survival of testicular grafts, which involves capillary exogenesis in grafts and large angiogenesis in recipients. Currently, testicular grafts are mostly avascular anastomosis tissues [5]. Evidence shows that the graft survival rate increased after encapsulating testicular tissues with alginate hydrogel and the adoption of vascular endothelial growth factor-encapsulated nanoparticles [19,24]. Therefore, we injected Matrigel into each incision to improve the survival and development of calf testicular grafts.

Excitingly, vascular networks around the xenografts were observed, and scattered capillaries were found in the interstitium of the grafts 28 d post-grafting, implying that circulation was established between the grafts and recipients. We also observed that the diameter of the seminiferous cords slightly increased and vacuoles appeared in the center of the seminiferous cords in the grafts, suggesting that the development of the seminiferous cords in the grafts is proceeding, and these vacuoles may be fused later to become the lumen of the seminiferous tubules. Spermatogenesis in bovine testis xenografts is less efficient [16,17] than that in mice [1,6], rabbits [6], pigs [1,7,8,9,10], goats [1], and sheep [13,14]. Generally, 4~8-week-old testis tissues were used for xenotransplantation in cattle [16,17,18,19] and buffalo [15]. Particularly, all these studies grafted fresh bovine testis tissues. Here, we xenografted cryopreserved 1D calf testis tissues using mouse recipients and established successful blood circulation between the survived grafts and the recipients post-grafting.

To further investigate xenograft development, the mRNA levels of germline genes and somatic genes were examined as reported [20,25]. The increased transcriptional expressions of germline gene *C-kit*, and somatic genes *Sox9* and *Star* in D28 grafts than those in normal D30 testis, suggest that the seminiferous epithelium and interstitial tissue in the xenografts are indeed developing and differentiating, which is consistent with the descriptions that spermatogenesis in most immature testicular grafts often shows a physiological status earlier than that in normal testes [26]. It is also noticed that the mRNA level of *Acta2* is not significantly higher than that in the D30 control; this could be due to our small sample number and relatively high standard deviation.

## 5. Conclusions

Here, we initially described cryopreserved calf testicular xenografting in immunocompromised mouse recipients. As an easy-to-prepare in vivo model, it is a useful tool to reveal testicular physiology and male germ cell development and preserve and protect the germplasm of humans, excellent breeding animals, as well rare and endangered species. The limitation of this brief report is that the grafts were grown only in a limited period due to the pandemic. The fate of seminiferous cords/tubules in the grafts needs to be observed for a longer amount of time in the future.

## Figures and Tables

**Table 1:** Primer sequences for qRT-PCR analysis.

Gene	Forward Primer (5′-3′)	Reverse Primer (5′-3′)
*GAPDH*	CGGCACAGTCAAGGCAGAGAAC	CGGCACAGTCAAGGCAGAGAAC
*GFRα-1*	TGGCCCTGCTTGTTTTCCTCT	ACAGGTATGCACGCTTGTGT
*C-Kit*	TGTCTGCACTGCTCAGCGAATC	TTGATGGCTGCCCGCACTTTC
*Sycp3*	CCGGGAAGTTGGCAAAACCA	GGCATCCTCCTCTGAACCACT
*Sox9*	AGGAGAGCGAGGAGGACAAGTTC	ACCAGCGTCCAGTCGTAGCC
*Acta2*	GATGGTGGGAATGGGACAGAAAGAC	GGTGATGATGCCGTGCTCTATCG
*Star*	AAGACCCTCTCTACAGCGACCAAG	GGATCACTTTACTCAGCACCTCGTC

## Data Availability

The data that support the findings of this study are available from the corresponding author upon reasonable request.
